# Validating the ACOSOG Z0011 Trial Result: A Population-Based Study Using the SEER Database

**DOI:** 10.3390/cancers12040950

**Published:** 2020-04-11

**Authors:** Jiwoong Jung, Byoung Hyuck Kim, Jongjin Kim, Sohee Oh, Su-jin Kim, Chang-Sup Lim, In Sil Choi, Ki-Tae Hwang

**Affiliations:** 1 Department of Surgery, Seoul Medical Center, Seoul 02053, Korea; jungjw@seoulmc.or.kr; 2 Department of Radiation Oncology, Seoul Metropolitan Government Seoul National University Boramae Medical Center, Seoul 07061, Korea; karlly71@hanmail.net; 3 Department of Surgery, Seoul Metropolitan Government Seoul National University Boramae Medical Center, Seoul 07061, Korea; michael5@hanmail.net (J.K.); limcs7@gmail.com (C.-S.L.); 4 Department of Biostatistics, Seoul Metropolitan Government Seoul National University Boramae Medical Center, Seoul 07061, Korea; oh.sohee@gmail.com; 5 Department of Surgery, Seoul National University College of Medicine, Seoul 03080, Korea; su.jin.kim.md@gmail.com; 6 Department of Internal Medicine, Seoul Metropolitan Government Seoul National University Boramae Medical Center, Seoul 07061, Korea; hmoischoi@hanmail.net

**Keywords:** axillary lymph node dissection, breast cancer, sentinel lymph node biopsy

## Abstract

The Z0011 trial demonstrated that axillary lymph node dissection (ALND) could be omitted in spite of 1–2 metastatic sentinel lymph nodes. This study aimed to validate the results on a population-based database. The Surveillance, Epidemiology, and End Results (SEER) database was searched for patients comparable to the Z0011 participants. The type of axillary surgery was estimated using the total number of examined axillary lymph nodes (ALNs). Breast cancer-specific mortality (BCSM) was compared between patients with ≥10 ALNs (the sentinel lymph node dissection (SLND) and ALND group, or “SLND + ALND group”) and patients with one or two ALNs (the “SLND group”). During 2010–2015, the SEER database included 7077 and 6620 patients categorized in the SLND group and the SLND + ALND group, respectively. Death was observed for 515 patients (7.3%) in the SLND group and 589 patients (8.9%) in the SLND + ALND group based on a median follow-up of 41 months. After propensity-score matching, the adjusted hazard ratio for BCSM in the SLND group (vs. the SLND + ALND group) was 1.038 (95% confidence interval: 0.798–1.350). Regardless of the SLND criteria, the outcomes were not significantly different between the two groups. This retrospective cohort study of Z0011-comparable patients revealed that ALND could be omitted based on the Z0011 strategy, even among patients with ≤2 dissected ALNs.

## 1. Introduction

Axillary management of breast cancer has evolved from conventional axillary lymph node dissection (ALND) to less invasive adjuvant therapies. For example, the American College of Surgeons Oncology Group (ACOSOG)’s Z0011 trial demonstrated that conventional ALND could be safely omitted without increasing recurrence or cancer-related death among clinically node-negative women with T1–T2 invasive breast cancer who were receiving breast-conserving surgery (BCT) with planned whole-breast irradiation and adequate systemic therapy, even if metastases were present in one or two sentinel lymph nodes (SLNs). The long-term Z0011 follow-up data also revealed non-inferior 10-year survival and loco-regional control outcomes for patients who underwent sentinel lymph node dissection (SLND) alone, relative to patients who underwent ALND [1,2]. Complications of ALND, such as pain, numbness, or lymphedema, might be prevented by omitting ALND.

The Z0011 results have led to a change in the standard axillary management of breast cancer [3,4,5], although they remain controversial for many conservative clinicians, based on the small sample size and short follow-up period (94 deaths from the entire 856 participants during a median follow-up of 6.3 years). The latter issue was partially resolved by the 10-year long-term follow-up data [1,2], although the sample size issue remains important (110 deaths from the entire 856 participants during a median follow-up of 9.3 years). Thus, the findings should be validated in a large Z0011-comparable cohort, which would enable sub-analyses for various conditions, such as a small number of total SLNs. In this context, only one or two SLN metastases were permitted for ALND omission, although that condition might be fulfilled without pathological confirmation if the patient only had one or two SLNs. Thus, as a small number of total SLNs does not guarantee a lower metastatic burden in the axillary nodes, the Z0011 strategy should be applied cautiously for those patients. The present study aimed to validate the Z0011 results within a large population-based Z0011-comparable cohort, and to investigate whether the Z0011 strategy is feasible among patients with ≤2 SLNs.

## 2. Results

### 2.1. Characteristics of the Z0011-Comparable SEER Cohort

The 23,138 Z0011-comparable SEER patients were assigned to the SLND group (7077 patients), the “SLND plus” group (9411 patients), and the SLND + ALND group (6620 patients) (Table 1). The median age was 60 years, the median tumor size was 1.8 cm, 86.1% of the patients had estrogen receptor (ER)-positive cancers, 58.1% of the patients received adjuvant chemotherapy, and 73.8% of the patients received radiation therapy (RT). The median follow-up was 41 months, and death was observed for 1760 patients (7.6%), including 865 breast cancer-specific mortality (BCSM) cases (3.7%). There was a significant increase in the ratio of SLND to SLND + ALND patients according to the year of diagnosis during 2010–2015.

### 2.2. Characteristics of the SLND Group

Among the Z0011-comparable SEER cohort, the SLND group had smaller and lower-grade tumors than the SLND + ALND group, as well as a higher ER-positive rate and a lower human epidermal growth factor receptor 2 (HER2) overexpression rate. The SLND group also had a far higher number of patients with only one positive LN. Adjuvant RT was slightly more common in the SLND group (74.9% vs. 71.8%; *p* < 0.001), while adjuvant chemotherapy was more common in the SLND + ALND group (49.0% vs. 69.7%; *p* < 0.001). The “SLND plus” group had characteristics that were intermediate between the SLND and SLND + ALND groups (Table 1).

Although the characteristics of the SLND group in the Z0011-comparable SEER cohort were generally similar to those in the original Z0011 cohort, adjuvant RT and chemotherapy were administered less frequently in the SEER cohort (Table S1). Furthermore, the SEER dataset only includes incomplete information regarding adjuvant therapies, which suggests that the actual rates of adjuvant therapy would be higher than our results.

### 2.3. Outcomes in the Z0011-Comparable Cohort

**Figure 1:**
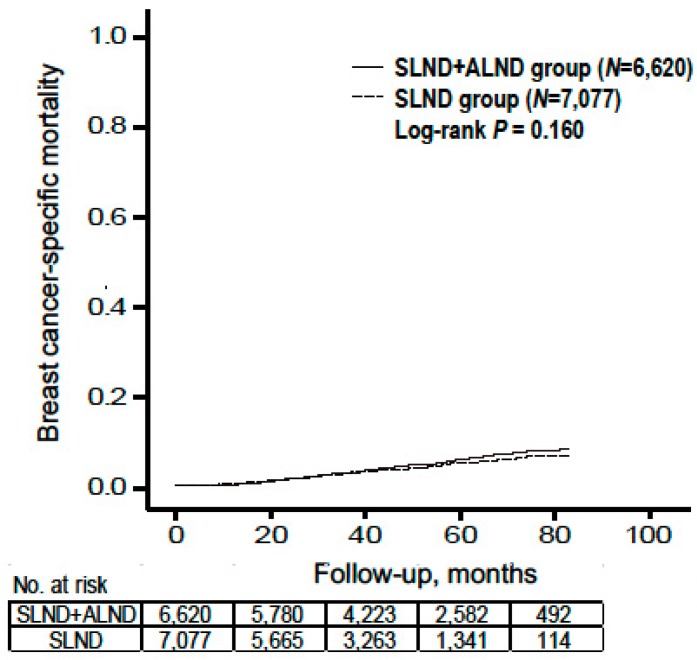
Breast cancer-specific mortality by type of axillary surgery. Abbreviations: axillary lymph node dissection (ALND); sentinel lymph node dissection (SLND).

The 865 BCSM cases (3.7%) included 219 patients (3.1%) in the SLND group and 312 patients (4.7%) in the SLND + ALND group. However, we omitted 334 patients (3.5%) in the “SLND plus” group because their axillary surgery type was unclear. The SLND group had a significantly shorter follow-up period, which might be related to the increasing proportion of the SLND cases (relative to SLND + ALND cases) during 2010–2015. We did not detect a significant difference in BCSM risk when we compared the SLND group to the SLND + ALND group (unadjusted hazard ratio (HR): 0.884, 95% confidence interval (CI): 0.743–1.051) (Figure 1). Univariate differences in BCSM were observed according to T category, histological grade and type, number of metastatic ALNs, node status, ER status, progesterone receptor (PR) status, chemotherapy status, and RT status. No significant differences in BCSM were observed according to age or HER2 status.

Furthermore, there was no significant difference in BCSM when we compared the SLND and SLND + ALND groups (adjusted HR: 1.065, 95% CI: 0.821–1.382) using a multivariable model (Table 2). The multivariable analysis revealed that BCSM was significantly influenced by T category, histological grade, age, number of metastatic ALNs, node status, ER status, PR status, HER2 status, chemotherapy status, and RT status. The effect of ALND omission on BCSM differed by the number of metastatic ALN(s) and RT status (interaction *p =* 0.008 and 0.023 respectively).

**Figure 2:**
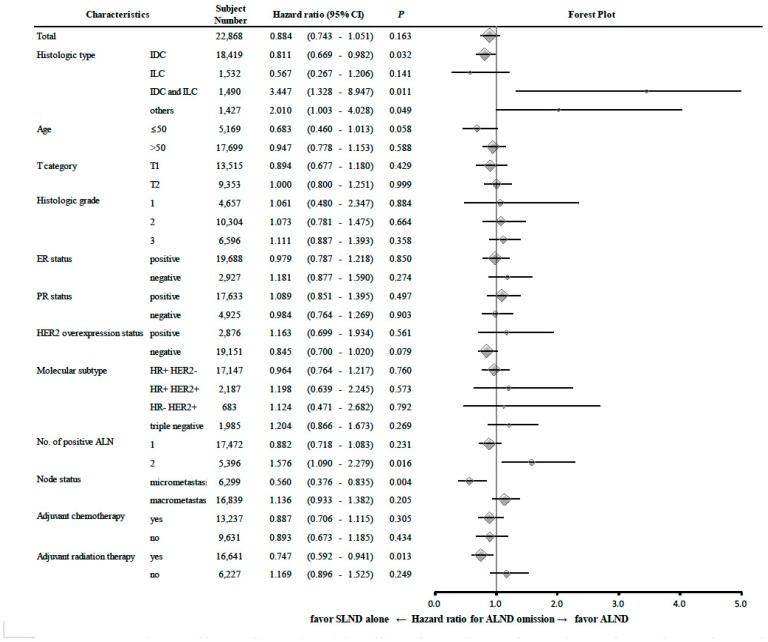
Risk of breast cancer-specific mortality in each subgroup of the Z0011-comparable patients according to the clinicopathologic risk factors. Abbreviations: axillary lymph node (ALN); axillary lymph node dissection (ALND); confidence interval (CI); estrogen receptor (ER); human epidermal growth factor receptor 2 (HER2); hazard ratio (HR); invasive ductal carcinoma (IDC); invasive lobular carcinoma (ILC); progesterone receptor (PR).

Subgroup analyses according to histological grade, age, T category, ER status, PR status, HER2 status, molecular subtype, node status and adjuvant therapy status revealed no significant difference in BCSM between the SLND and SLND + ALND groups (Figure 2). Among patients with two metastatic ALNs, the SLND group had a higher BCSM rate than the SLND + ALND group (HR: 1.576, 95% CI: 1.090–2.279). Interestingly, both ALNs were metastatic for all SLND patients with two ALNs in total.

### 2.4. Comparing BCSM after PS Matching

We performed propensity score (PS) matching to reduce bias related to the influence of patient and tumor characteristics on the decision to omit ALND. After omitting 422 patients in the SLND group and 424 patients in the SLND + ALND group because of missing data, PS matching was performed for 6655 patients in the SLND group and 6196 patients in the SLND + ALND group. The PSs were calculated using a logistic regression model with the following independent variables: age (≤50 vs. >50 years), T category, number of positive ALN(s), micro-/macro-metastasis, histological type and grade, ER status, PR status, HER2 status, and adjuvant therapy status. In total, we identified 7194 PS-matched patients (3597 patients in the SLND group, 3597 patients in the SLND + ALND group). The standardized differences before and after the PS matching are summarized in Table S2.

**Figure 3:**
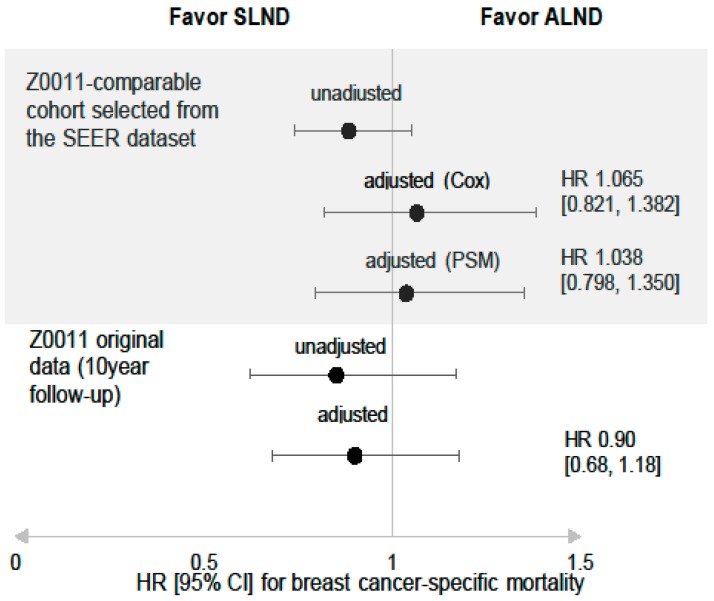
Adjusted hazard ratios of the SLND group to the SLND + ALND group, for breast cancer-specific mortality after propensity score matching. Abbreviations: axillary lymph node (ALN); axillary lymph node dissection (ALND); confidence interval (CI); hazard ratio (HR); sentinel lymph node dissection (SLND).

Among the 7194 PS-matched patients, the adjusted HR for BCSM in the SLND group was 1.038 (95% CI: 0.798–1.350). This result agrees with the result from the multivariable analysis and supports the Z0011 strategy. Figure 3 shows our adjusted HR and the reported HR for BCSM from the Z0011 trial [2].

### 2.5. Sensitivity Analysis of BCSM after PS Matching

We also performed sensitivity analyses based on the possibility that patients would be unintentionally excluded from the SLND group based on our operational definition. The SLND group was redefined using one, three or four as the total number of ALNs, and the BCSM outcomes were compared between those groups and the SLND + ALND group after PS matching. None of the ALN criteria provided a significant difference in BCSM between the SLND and SLND + ALND groups (Figure S1).

## 3. Discussion

To validate the Z0011 strategy using the SEER dataset, we operationally defined the SLND group as having ≤2 ALNs. This allowed us to select patients who underwent SLND alone, with minimal inclusion of patients who underwent conventional ALND, as SLND removes an average of two LNs in most studies [6,7,8]. However, relative to patients who truly underwent SLND alone, patients in our SLND group might be more susceptible to recurrence and/or cancer-related mortality, given the relative high possibility of a false negative SLND result using a small number of ALNs [9]. Our operational definition is also useful because patients in the SLND group are unique in that their SLND pathological results have little significance in determining whether to proceed with conventional ALND under the Z0011 strategy, as the number of positive SLNs could not exceed two. Thus, the applicability of the Z0011 results within that group is a clinically significant issue that needed to be validated separately.

Another retrospective study has evaluated an Asian Z0011-eligible cohort, and confirmed that ALND omission did not increase the risk of recurrence, even among patients with ≤2 SLNs [10]. However, that study did not evaluate cancer-related deaths because of limited information regarding mortality. In contrast, the present study involved a median follow-up of 41 months and identified 865 BCSM cases (3.7%) and 1760 cases (7.6%) of all-cause mortality. The present study also involved a large number of subjects, which allowed for a useful survival analysis despite the short follow-up period. Finally, the present study’s mortality rate was similar to the Z0011 results, based on 5-year overall survival rates of 92.5% in the SLND-alone group and 91.8% in the ALND group [11].

Conventional ALND is thought to require ≥10 LNs to provide an adequate axillary assessment during breast cancer staging, and we used that cut-off to define our SLND + ALND group. These patients had only between zero and one metastatic nodes identified via further ALND after the one or two SLN metastases were detected, which indicates they are a unique subgroup with less metastatic burden than patients with ≥2 additional nodal metastases going through the same process, and that they would experience more favorable outcomes. However, the SLND patients might have poorer outcomes than patients with ≥3 SLNs, based on their higher possibility of false negative results. Thus, the lack of a significant difference in BCSM between the SLND and ALND + SLND groups supports the Z0011 strategy, and demonstrates that a small number of SLNs (e.g., ≤2 SLNs) had a minimal effect on BCSM when using the Z0011 strategy.

Our operational definition assumed that SLND was performed for all patients who had one or two ALN metastases in the final pathological report. Thus, the SLND + ALND group might have included some patients with upfront ALND but without SLND, based on the results of image-guided cytology. However, these patients all had small tumors and a low nodal metastatic burden, which suggests that their outcomes would be similar regardless of whether the metastatic LNs were detected via SLND or image-guided cytology. In addition, the initial method of detecting metastatic LNs would have little influence on patient outcomes if their nodal status is fixed based on the final pathological examination. That assumption is parallel to the previous attempts to expand the Z0011 strategy based on preoperative imaging and/or image-guided cytology [12,13,14,15,16].

The present study’s retrospective design might have introduced biases that disguised poorer outcomes in the SLND group, relative to the SLND + ALND group, especially as we detected differences between the two groups that suggested that selection bias affected the decision to omit ALND (Table 1). Thus, we performed PS matching and re-assessed the outcomes, which failed to reveal a significant difference in BCSM between the SLND and SLND + ALND groups. We believe that this approach is useful for overcoming the limitations of a retrospective validation study, especially given that a prospective validation of the Z0011 trial results would not be feasible, given the availability of long-term follow-up data and several previous validation studies.

Interestingly, among patients with two ALN metastases, the SLND group had a significantly higher BCSM rate, relative to the SLND + ALND group (Figure 2), although we suspect that this difference might have been intensified by our operational grouping. In this context, SLND patients with two ALN metastases might have a higher possibility of additional metastatic nodes that would be identified via further ALND, relative to patients with metastasis-free SLNs, which could be related to the ordinal position of the first positive LN among all removed SLNs. Yi et al. [17] have demonstrated that the first metastatic SLN was found to be the “hottest” SLN during SLND in 69% of their cases, and that the likelihood of metastatic disease decreased with each successive SLN that was evaluated. Thus, given that the SLN is the first in a regional lymphatic basin to accept drainage from the primary tumor [18,19,20], latter SLNs would be less likely to be informative. Therefore, it might be prudent to cautiously apply the Z0011 strategy for patients with metastases in 100% of a small number of SLNs.

This study has several limitations. First, the median follow-up was relatively short, given the outcomes of early-stage and ER-positive tumors, which highlights the importance of prolonged follow-up with an analysis of late events. Second, the SEER database does not include detailed information regarding endocrine therapy or specific RT fields, such as high-tangential or nodal irradiation, which precludes related analyses. However, evaluating patients from the post-Z0011 era presumably means that most patients received adequate endocrine therapy, and the roles of specific RT fields in the Z0011 results remain unclear. Furthermore, we failed to detect significant differences in the adjusted HRs for BCSM from the multivariable and PS-matched analyses (Table 2 and Figure 3), which suggests that any potential bias is unlikely to change our findings. Third, possible caveats associated with having two very different patient populations, as shown in Table 1, should be still considered, although we calibrated those parameters with statistical methods.

This study’s major strength is the use of the largest Z0011-comparable cohort from a population-based database, although the potential influence of selection bias should not be overlooked. Furthermore, to overcome the lack of information regarding axillary surgery type, we used an operational definition based on clinical experience and logical deduction. Moreover, our results might be meaningful in evaluating the Z0011 strategy in a contemporary cohort, although additional information is needed regarding late outcomes.

## 4. Materials and Methods

### 4.1. SEER Database and Cases

**Figure 4:**
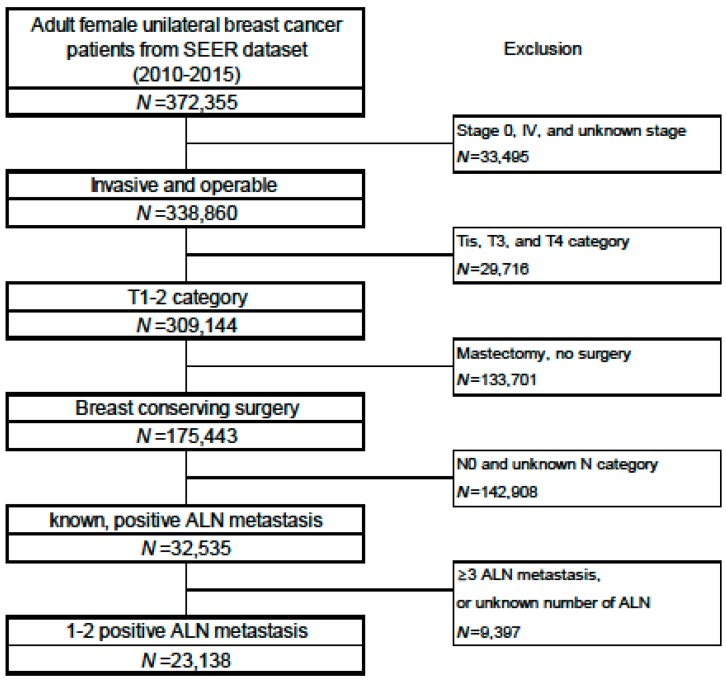
Subject selection process from the SEER database. Abbreviations: axillary lymph node (ALN).

The Surveillance, Epidemiology, and End Results (SEER) database [21] is maintained by the US National Cancer Institute and covers 18 population-based registries from 1973 to 2016 (approximately 30% of American patients). We retrospectively identified Z0011-comparable patients during 2010–2015 using SEER*Stat 8.3.6 software. The retrospective search identified 23,138 women with T1–T2 invasive breast cancer, primary BCT, and one or two metastatic axillary lymph nodes (ALNs) (Figure 4).

**Figure 5:**
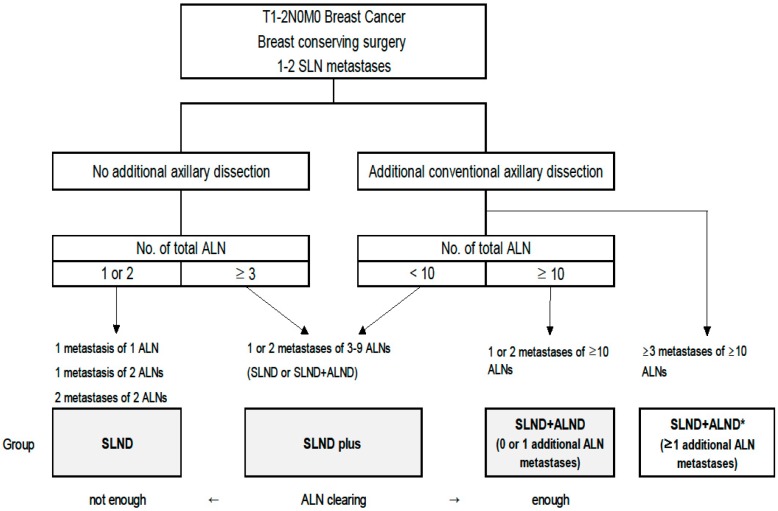
Assumed decision flow for patients who were eligible to the Z0011 strategy. Abbreviations: axillary lymph node (ALN); axillary lymph node dissection (ALND); sentinel lymph node (SLN); sentinel lymph node dissection (SLND). * Patients who had ≥3 metastases were not included in this study.

The SEER dataset did not specify the type of axillary surgery, which we operationally defined as SLND and/or ALND based on a set of three assumptions (Figure 5): 1) all 23,138 patients underwent SLND and/or ALND, 2) most patients with one or two examined ALNs underwent SLND alone, and 3) most patients with ≥10 ALNs underwent conventional ALND based on their SLND results. Based on those assumptions, we assigned patients with one or two examined ALNs to the SLND alone group (the “SLND group”), and patients with ≥10 examined ALNs to the “SLND + ALND group”. Patients with three to nine ALNs were assigned to an “SLND plus group” because we could not determine whether they underwent SLND alone or SLND + ALND.

The primary outcome was defined as breast cancer-specific mortality (BCSM), based on a “breast”-related cause in the SEER dataset. Deaths from other causes were assumed to be censored at the time of death.

### 4.2. Sensitivity Analysis

The total number of lymph nodes acquired after SLND could be only one or ≥3 in the real world. Thus, the outcomes of “SLND alone” patients might be different from the outcomes in our “SLND group”. To address that potential discrepancy, we performed a sensitivity analysis with other criteria for defining the SLND group based on the number of total ALNs (one, three and four ALNs). The corresponding risk estimates were calculated in the same way as the main analysis.

### 4.3. Propensity Score Matching

The effects of selection bias were minimized by matching propensity scores (PSs), which were calculated using a logistic regression model with the SLND group as the dependent variable and other variables that were selected based on their univariate associations with the SLND group. the logistic regression model for PS calculation included the following independent variables: age (≤50 vs. >50 years), T category, number of positive ALN(s), micro-/macro-metastasis, histologic type and grade, ER status, PR status, HER2 status, and adjuvant therapy status. Patients from the SLND and SLND + ALND groups were paired 1:1 using nearest-neighbor matching with a caliper width less than 0.25 standard deviations. Standardized differences were estimated before and after the matching to evaluate the covariates’ balance, with absolute values of <0.1 considered indicative of well-balanced groups [22,23]. These analyses were performed with R software version 3.5.2.

### 4.4. Statistical Analyses

The two groups’ characteristics were compared using the chi-squared test and two-sample t-tests. Survival curves were compared using the Kaplan–Meier method and log-rank test. Cox’s proportional hazard regression models were used to calculate hazard ratios (HRs) and 95% confidence intervals (CIs) for the associations of BCSM with the prognostic variables and treatments. All tests were two-sided and *p*-values of ≤0.05 were considered statistically significant. The analyses were performed using IBM SPSS software (version 20.0; IBM Corp., Armonk, NY, USA) and SAS software (version 9.3; SAS Institute, Cary, NC, USA).

## 5. Conclusions

This retrospective study of Z0011-comparable patients from the SEER database revealed that ALND could be omitted without increasing the risk of BCSM, based on a median follow-up of 41 months. Furthermore, our results suggest that the small number of SLNs had a minimal effect on the risk of BCSM based on the Z0011 strategy.

## Figures and Tables

**Table 1:** Baseline characteristics of the Z0011-compable patients selected from the Surveillance, Epidemiology, and End Results (SEER) database.

Characteristis	Total	SLND (ALN 1–2)	SLND Plus (ALN 3–9)	SLND + ALND (ALN ≥10)	*p*-Value
	No. (%)	No. (%)	No. (%)	No. (%)	
Subject number	23,138	7077	9441	6620	
Year					<0.001
2010	3614	595	1157	1862	
2011	3831	1051	1524	1256	
2012	3731	1227	1515	989	
2013	3841	1281	1678	882	
2014	3986	1423	1708	855	
2015	4135	1500	1859	776	
Patient age, years (range)	60 (20–101)	62 (23–98)	60 (24–101)	59 (20–97)	<0.001
≤50	5227 (22.6)	1300 (18.4)	2146 (22.7)	1781 (26.9)	
>50	17,911 (77.4)	5777 (81.6)	7295 (77.3)	4839 (73.1)	
Tumor size, median (cm)	1.8	1.8	1.8	2.0	<0.001
T category					<0.001
T1	13,674 (59.1)	4448 (62.9)	5641 (59.8)	3585 (54.2)	
T2	9464 (40.9)	2629 (37.1)	3800 (40.2)	3035 (45.8)	
No. of positive ALN(s)					<0.001
1	17,677 (76.4)	6439 (91.0)	7029 (74.5)	4209 (63.6)	
2	5461 (23.6)	638 (9.0)	2412 (25.5)	2411 (36.4)	
Histologic type					<0.001
IDC	18,643 (80.6)	5538 (78.3)	7631 (80.8)	5474 (82.7)	
ILC	1545 (6.7)	562 (7.9)	659 (7.0)	324 (4.9)	
IDC and ILC	1508 (6.5)	510 (7.2)	618 (6.5)	380 (5.7)	
others	1442 (6.2)	467 (6.6)	533 (5.6)	442 (6.7)	
Histologic grade					<0.001
1	4316(18.7)	1536 (21.7)	1830 (19.4)	950 (14.4)	
2	10,849 (46.9)	3523 (49.8)	4492 (47.6)	2834 (42.8)	
3	7452 (32.2)	1846 (26.1)	2913 (30.9)	2693 (40.7)	
unknown	521 (2.3)	172 (2.4)	206 (2.2)	143 (2.2)	
ER status					<0.001
positive	19,927 (86.1)	6365 (89.8)	8184 (86.7)	5378 (81.2)	
borderline	5 (0.0)	1 (0.0)	2 (0.0)	2 (0.0)	
negative	2957 (12.8)	634 (9.0)	1160 (12.3)	1163 (17.6)	
unknown	249 (1.1)	77 (1.1)	95 (1.0)	77 (1.2)	
PR status					<0.001
positive	17,845 (77.1)	5760 (81.4)	7404 (78.4)	4681 (70.7)	
borderline	26 (0.1)	7 (0.1)	9 (0.1)	9 (0.1)	
negative	4982 (21.5)	1224 (17.3)	1920 (20.3)	1838 (27.8)	
unknown	285 (1.2)	86 (1.2)	108 (1.1)	91 (1.4)	
HER2 status					<0.001
positive	2917 (12.6)	699 (9.9)	1184 (12.5)	1034 (15.6)	
borderline	433 (1.9)	133 (1.9)	177 (1.9)	123 (1.9)	
negative	19,377 (83.7)	6130 (86.6)	7922 (83.9)	5325 (80.4)	
unknown	411 (1.8)	115 (1.6)	158 (1.7)	138 (2.1)	
Molecular subtype					<0.001
HR+, HER2−	17,354 (75.0)	5691 (80.4)	7141 (75.6)	4522 (68.3)	
HR+, HER2+	2220 (9.6)	546 (7.7)	897 (9.5)	777 (11.7)	
HR−, HER2+	691 (3.0)	153 (2.2)	286 (3.0)	252 (3.8)	
triple negative	2004 (8.7)	431 (6.1)	772 (8.2)	801 (12.1)	
unknown	869 (3.8)	256 (3.6)	345 (3.7)	268 (4.0)	
Adjuvant therapy					
chemotherapy	13,449 (58.1)	3467 (49.0)	5367 (56.8)	4615 (69.7)	<0.001
radiation therapy	17,082 (73.8)	5299 (74.9)	7033 (74.5)	4750 (71.8)	<0.001
Median follow-up, months (IQR)	41 (24–61)	37 (22–55)	39 (23–58)	50 (30–70)	
Deaths	1760 (7.6)	515 (7.3)	656 (6.9)	589 (8.9)	
breast cancer	865 (3.7)	219 (3.1)	334 (3.5)	312 (4.7)	
other cause	895 (3.9)	296 (4.2)	322 (3.4)	277 (4.2)	

Abbreviations: Abbreviations: axillary lymph node (ALN); axillary lymph node dissection (ALND); confidence interval (CI); estrogen receptor (ER); human epidermal growth factor receptor 2 (HER2); hazard ratio (HR); invasive ductal carcinoma (IDC); invasive lobular carcinoma (ILC); progesterone receptor (PR); sentinel lymph node dissection (SLND).

**Table 2:** Multivariable analysis for breast cancer-specific mortality among the Z0011-comparable patients.

Variable	Univariable Analysis		Multivariable Analysis ^a^	
	HR (95% CI)	*p*-Value	HR (95% CI)	*p*-Value
Extent of axillary clearance				
SLND vs. SLND + ALND	0.884 (0.743–1.051)	0.163	1.065 (0.821–1.382)	0.636
Age				
>50 vs. ≤50	1.052 (0.897–1.234)	0.532		
No. of metastatic ALN(s)				
2 vs. 1	1.335 (1.154–1.545)	<0.001	1.285 (1.025–1.611)	0.030
Node status				
macro- vs. micro-metastasis	1.534 (1.300–1.811)	<0.001	1.279 (1.076–1.521)	0.005
T category				
T2 vs. T1	2.469 (2.152–2.832)	<0.001	1.856 (1.612–2.137)	<0.001
Histologic grade				
2 vs. 1	2.132 (1.571–2.893)	<0.001	1.800 (1.323–2.449)	<0.001
3 vs. 1	6.360 (4.746–8.522)	<0.001	3.463 (2.534–4.733)	<0.001
Histologic type				
ILC vs. IDC	0.747 (0.551–1.012)	0.060	1.098 (0.802–1.504)	0.559
IDC and ILC vs. IDC	0.664 (0.481–0.916)	0.013	0.938 (0.676–1.300)	0.699
others vs. IDC	0.924 (0.699–1.221)	0.579	0.857 (0.648–1.133)	0.278
ER status				
positive vs. negative	0.248 (0.216–0.285)	<0.001	0.618 (0.505–0.758)	<0.001
PR status				
positive vs. negative	0.274 (0.239–0.313)	<0.001	0.509 (0.419–0.618)	<0.001
HER2 status				
positive vs. negative	1.043 (0.856–1.272)	0.676		
Adjuvant chemotherapy				
no vs. yes	0.869 (0.757–0.998)	0.047	1.492 (1.284–1.734)	<0.001
Adjuvant radiotherapy				
no vs. yes	1.948 (1.699–2.233)	<0.001	1.493 (1.186–1.881)	0.001
No. of metastatic ALN(s) ×Extent of axillary clearance (2 vs. 1)				0.008 ^b^
SLND vs. SLND + ALND			1.594 (1.046–2.429)	0.030
Adjuvant radiotherapy×Extent of axillary clearance (no vs. yes)				0.023 ^b^
SLND vs. SLND + ALND			1.418 (0.998–2.014)	0.052

Abbreviations: axillary lymph node (ALN); axillary lymph node dissection (ALND); confidence interval (CI); estrogen receptor (ER); human epidermal growth factor receptor 2 (HER2); hazard ratio (HR); invasive ductal carcinoma (IDC); invasive lobular carcinoma (ILC); progesterone receptor (PR); sentinel lymph node dissection (SLND). ^a^ Variables with *p* < 0.1 in univariable analyses and the extent of axillary clearance were inputted into multivariable analysis. ^b^ Interaction *p*-values.

## Supplementary Materials

The following are available online at https://www.mdpi.com/2072-6694/12/4/950/s1. Figure S1: Adjusted hazard ratios of the SLND group to the SLND + ALND group, for breast cancer-specific mortality after propensity score matching, Table S1: Comparison between the Z0011-comparable cohort in this study and the ACOSOG Z0011 study cohort, Table S2: Standardized differences before and after propensity score matching for the Z0011-comparable cohort.
