# Supplementary material for: Validating the ACOSOG Z0011 Trial Result: A Population-Based Study Using the SEER Database
Source: Cancers (Basel). 2020 Apr 11;12(4):950. doi: 10.3390/cancers12040950 (PMC7226449; doi:10.3390/cancers12040950)
Supplement: Supplementary file 1 [file cancers-12-00950-s001.pdf]

Supplementary Material: Validating the ACOSOG Z0011 Trial Result: A Population-Based Study Using the SEER Database

Jiwoong Jung, Byoung Hyuck Kim, Jongjin Kim, Sohee Oh, Su-jin Kim, Chang-Sup Lim, In Sil Choi and Ki-Tae Hwang

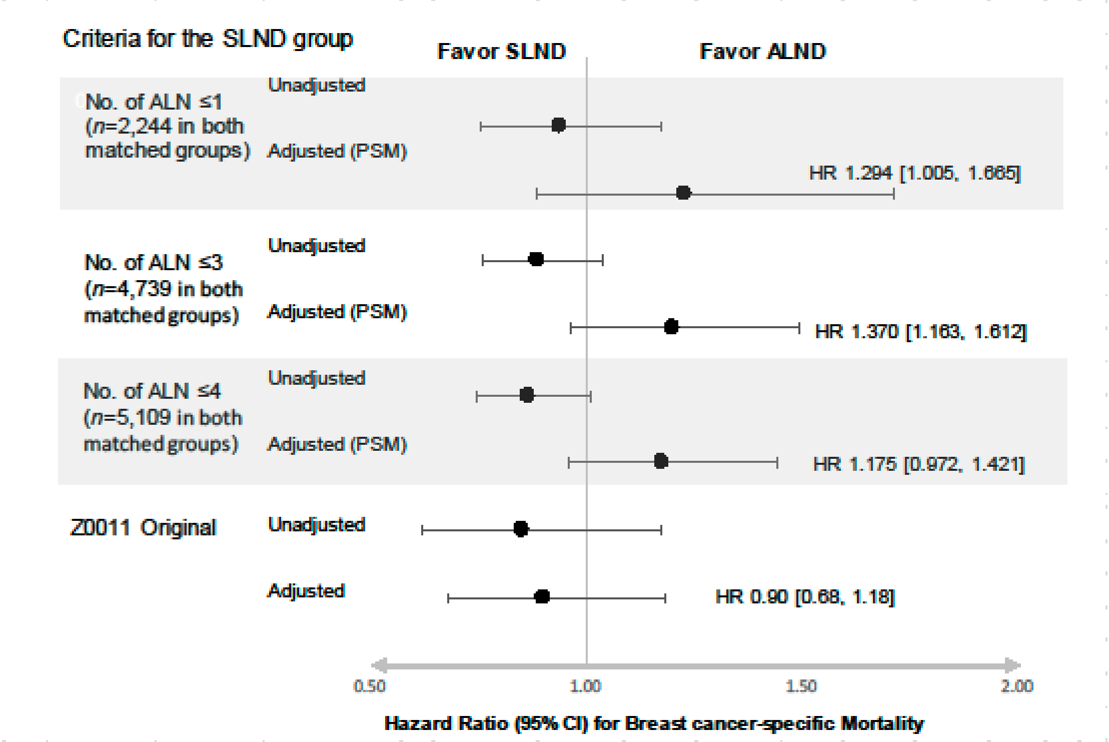

Figure S1. Adjusted hazard ratios of the SLND group to the SLND+ALND group, for breast cancer-specific mortality after propensity score matching. Abbreviations: ALND, axillary lymph node dissection; CI, confidence interval; HR, hazard ratio; SLND, sentinel lymph node dissection

Table S1. Comparison between the Z0011-comparable cohort in this study and the ACOSOG Z0011 study cohort.

Characteristics	Total	SLND (ALN 1–2)	ACOSOG Z0011 (Intention-to-Treat Sample)	
			SLND Alone	ALND
	No. (%)	No. (%)	No. (%)	No. (%)
Subject number	23,138	7,077	436	420
Patient age, years (range)	60 (20–101)	62 (23–98)	54 (25–90)	56 (24–92)
≤50	5,227 (22.6)	1,300 (18.4)	160 (36.7)	135 (32.1)
>50	17,911 (77.4)	5,777 (81.6)	266 (61.0)	278 (66.2)
missing			10	7
Tumor size, mean (cm)	1.8	1.8	1.6	1.7
T1	13,674 (59.1)	4,448 (62.9)	303 (70.6)	284 (67.9)
T2	9,464 (40.9)	2,629 (37.1)	126 (29.4)	134 (32.1)
N category				
N1	23,138 (100.0)	7,077 (100.0)	382 (92.1)	292 (85.1)
N2-3			4 (1.0)	47 (13.7)

missing			21	77
Number of positive SLN				
0			29 (7.0)	4 (1.2)
1	17,677 (76.4)	6,439 (91.0)	295 (71.1)	199 (58.0)
2	5,461 (23.6)	638 (9.0)	76 (18.3)	68 (19.8)
≥3			15 (3.6)	72 (21.0)
missing			21	77
Histologic grade				
1	4,316(18.7)	1,536 (21.7)	81 (25.6)	71 (22.0)
2	10,849 (46.9)	3,523 (49.8)	148 (46.8)	158 (48.9)
3	7,452 (32.2)	1,846 (26.1)	87 (27.5)	94 (29.1)
missing	521 (2.3)	172 (2.4)	120	97
ER status				
positive	19,927 (86.1)	6,365 (89.8)	324 (82.7)	317 (82.7)
borderline	5 (0.0)	1 (0.0)		
negative	2,957 (12.8)	634 (9.0)	68 (17.3)	66 (17.3)
missing	249 (1.1)	77 (1.1)	44	37
PR status				
positive	17,845 (77.1)	5,760 (81.4)	274 (69.9)	259 (67.6)
borderline	26 (0.1)	7 (0.1)		
negative	4,982 (21.5)	1,224 (17.3)	118 (30.1)	124 (32.4)
missing	285 (1.2)	86 (1.2)	44	37
Histologic type				
IDC	18,643 (80.6)	5,538 (78.3)	356 (84.0)	344 (82.7)
ILC	1,545 (6.7)	562 (7.9)	36 (8.5)	27 (6.5)
IDC and ILC	1,508 (6.5)	510 (7.2)	-	-
others	1442 (6.2)	467 (6.6)	32 (7.5)	45 (10.8)
missing			12	4
Adjuvant treatment				
chemotherapy	13,449 (58.1)	3,467 (49.0)	253 (58.0)	243 (57.9)
radiation therapy	17,082 (73.8)	5,299 (74.9)	277 (89.6)	263 (88.9)
endocrine therapy			203 (46.6)	195 (46.4)
Median follow-up, months (IQR)	41 (24–61)	37 (22–55)	75 (62–92)	
Deaths	1760 (7.6)	515 (7.3)	42 (9.6)	52 (12.4)
breast cancer	865 (3.7)	219 (3.1)		
other cause	895 (3.9)	296 (4.2)		

Abbreviations: ALND, axillary lymph node dissection; ER, estrogen receptor; IDC, invasive ductal carcinoma; ILC, invasive lobular carcinoma; PR, progesterone receptor; SLN, sentinel lymph node; SLND, sentinel lymph node dissection

Table S2. Standardized differences before and after propensity score matching for the Z0011-comparable cohort.

Variables	Before Matching		SD	After Matching		SD
	SLND + ALND	SLND		SLND + ALND	SLND	
	(*N* = 6,196)	(*N* = 6,655)		(*N* = 3,597)	(*N* = 3,597)	
**Age**			0.0862			−0.0033
>50	1666 (26.9%)	1216 (18.3%)		739 (20.5%)	751 (20.9%)	
≤50	4530 (73.1%)	5439 (81.7%)		2858 (79.5%)	2846 (79.1%)	
Histologic type			0.0535			0.0078
IDC	5135 (82.9%)	5240 (78.7%)		3016 (83.8%)	3006 (83.6%)	
ILC	301 (4.9%)	506 (7.6%)		201 (5.6%)	203 (5.6%)	
IDC and ILC	357 (5.8%)	488 (7.3%)		195 (5.4%)	193 (5.4%)	
Others	403 (6.5%)	421 (6.3%)		185 (5.1%)	195 (5.4%)	
Histologic grade			−0.2283			−0.0081
1	910 (14.7%)	1492 (22.4%)		622 (17.3%)	627 (17.4%)	
2	2707 (43.7%)	3398 (51.1%)		1768 (49.2%)	1787 (49.7%)	
3	2579 (41.6%)	1765 (26.5%)		1207 (33.6%)	1183 (32.9%)	
T category			−0.0882			0.0053
T1	3359 (54.2%)	4195 (63.0%)		2125 (59.1%)	2106 (58.5%)	
T2	2837 (45.8%)	2460 (37.0%)		1472 (40.9%)	1491 (41.5%)	
Adjuvant radiation therapy			0.0298			0.0025
No	1682 (27.1%)	1608 (24.2%)		926 (25.7%)	917 (25.5%)	
Yes	4514 (72.9%)	5047 (75.8%)		2671 (74.3%)	2680 (74.5%)	
Node status			0.2798			0.0011
macrometastasis	5440 (87.8%)	3981 (59.8%)		2995 (83.3%)	2991 (83.2%)	
micrometastasis	756 (12.2%)	2674 (40.2%)		602 (16.7%)	606 (16.8%)	
Adjuvant chemotherapy			−0.2102			0.0011
No	1840 (29.7%)	3375 (50.7%)		1310 (36.4%)	1306 (36.3%)	
Yes	4356 (70.3%)	3280 (49.3%)		2287 (63.6%)	2291 (63.7%)	

No. of Positive ALN			−0.2715			0.0017
1	3945 (63.7%)	6044 (90.8%)		3077 (85.5%)	3071 (85.4%)	
2	2251 (36.3%)	611 (9.2%)		520 (14.5%)	526 (14.6%)	
ER status			0.0891			-0.0011
Negative	1108 (17.9%)	597 (9.0%)		433 (12.0%)	437 (12.1%)	
Positive	5088 (82.1%)	6058 (91.0%)		3164 (88.0%)	3160 (87.9%)	
PR status			0.1083			−0.0058
Negative	1749 (28.2%)	1158 (17.4%)		729 (20.3%)	750 (20.9%)	
Positive	4447 (71.8%)	5497 (82.6%)		2868 (79.7%)	2847 (79.1%)	
HER2 status			−0.0582			−0.0131
Negative	5203 (84.0%)	5976 (89.8%)		3170 (88.1%)	3217 (89.4%)	
Positive	993 (16.0%)	679 (10.2%)		427 (11.9%)	380 (10.6%)	

Abbreviations: ALN, axillary lymph node; ALND, axillary lymph node dissection; ER, estrogen receptor; HER2, human epidermal growth factor receptor 2; IDC, invasive ductal carcinoma; ILC, invasive lobular carcinoma; PR, progesterone receptor; SD, standardized difference; SLND, sentinel lymph node dissection

© 2020 by the authors. Licensee MDPI, Basel, Switzerland. This article is an open access article distributed under the terms and conditions of the Creative Commons Attribution (CC BY) license (http://creativecommons.org/licenses/by/4.0/).
